# An incidental finding of large cell calcifying Sertoli cell tumor in an orchidectomy specimen for a separate adenomatoid tumor

**DOI:** 10.1002/iju5.12626

**Published:** 2023-09-20

**Authors:** Lauren Crone, Sophie C Prendergast, Nick Mayer, Frank O'Brien

**Affiliations:** ^1^ Department of Urology Royal College of Surgeons of Ireland Dublin Ireland; ^2^ Department of Histopathology Cork University Hospital Cork Ireland; ^3^ Department of Urology Cork University Hospital Cork Ireland

**Keywords:** adenomatoid tumor, incidental tumor, large cell calcifying Sertoli cell tumor, orchidectomy, testicular calcifications

## Abstract

**Introduction:**

Large cell calcifying Sertoli cell tumors are exceedingly rare. They are most commonly benign, but risks for malignancy include older age, larger size of tumor, and solitary tumors. To the author's knowledge, this is the first case reported of an incidental large cell calcifying Sertoli cell tumor when an orchidectomy was performed for a separate lesion.

**Case presentation:**

A 31‐year‐old male presented with a painless testicular lump. Ultrasound demonstrated an exophytic lesion in the superolateral aspect and calcifications were noted inferomedially and inferolaterally in the right testis. On histology from radical orchidectomy, the superolateral lesion was found to be an adenomatoid tumor, and the calcifications inferiorly represented a large cell calcifying Sertoli cell tumor. The background showed foci of germ cell neoplasia in situ but no evidence of invasive malignancy.

**Conclusion:**

Calcifications on ultrasound in isolation may represent large cell calcifying Sertoli Cell tumors.

**Abbreviations & Acronyms**
LCCSC: Large cell calcifying Sertoli cell

**Keynote message**

In this paper, we describe a rare tumor, picked up as an incidental finding on a pathology specimen for a separate neoplasm. To our knowledge, this is the first described case of an incidental finding of a large cell calcifying Sertoli cell tumor. Given the paucity of knowledge on these tumors, this case report will be valuable for clinicians in evaluating the clinical implications of ultrasound findings.

## Introduction

LCCSC tumors are rare testicular neoplasms and 80% are benign. They can be associated with the genetic disorders Peutz‐Jager and Carney's complex.^1^ This is the first reported case of an LCCSC tumor as an incidental finding when a testicle was removed for a separate indication. In this case, preoperative ultrasound failed to distinguish the LCCSC tumor.

## Case presentation

This 31‐year‐old was referred to the urology clinic with a hard lesion on the superior aspect of his right testis noticed on self‐examination 4 weeks prior. Otherwise, he was clinically well, on no regular medication, and with no family history of note. He had no history to suggest a genetic disorder. On examination, there was a 1 cm hard lesion extending from the upper pole of the right testis. This was well‐circumscribed, non‐tender, and partially exophytic. The rest of his examination was normal.

**Fig. 1:**
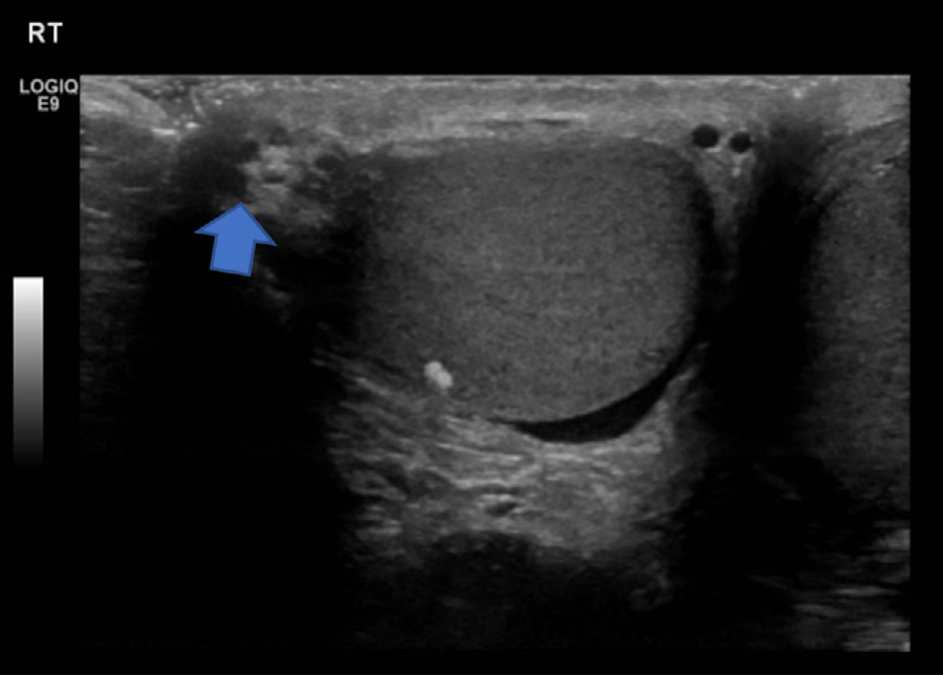
Right testicular ultrasound showing a heterogenous lesion at the superior pole of the testis (arrow).

**Fig. 2:**
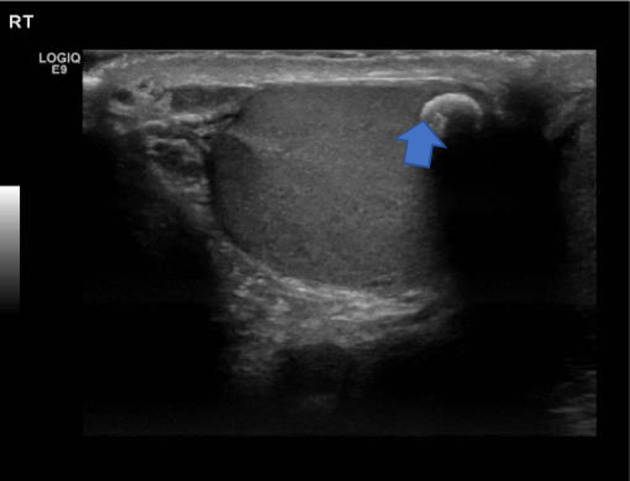
Right testicular ultrasound showing a large calcification at the inferior aspect of the testis (arrow).

Alpha‐fetoprotein, lactate dehydrogenase, and beta‐human chorionic gonadotrophin were normal. An ultrasound showed a partially exophytic soft tissue nodular lesion arising from the superolateral aspect of the right testis (Fig. 1). This was heterogenous in echotexture and demonstrated central vascularity. It measured 0.8 × 0.7 × 0.8 cm. This corresponded to the hard mass felt on the exam, and seemed to be arising from within the testes. There was an additional 0.7 cm area of coarse calcification inferomedially in the right testis with focal parenchymal (Fig. 2).

The sonographic features of the lesion on the superior aspect of the right testis were strongly suspicious of a testicular malignancy. This was based on the heterogenous echotexture and central vascularity. The right lower pole testicular calcification present was favored to be post‐traumatic or post‐inflammatory in etiology and did not affect the management plan. A radical inguinal orchidectomy was performed. On the day of his operation, he underwent a contrast‐enhanced computerized tomography of the thorax abdomen, and pelvis which excluded metastatic disease.

**Fig. 3:**
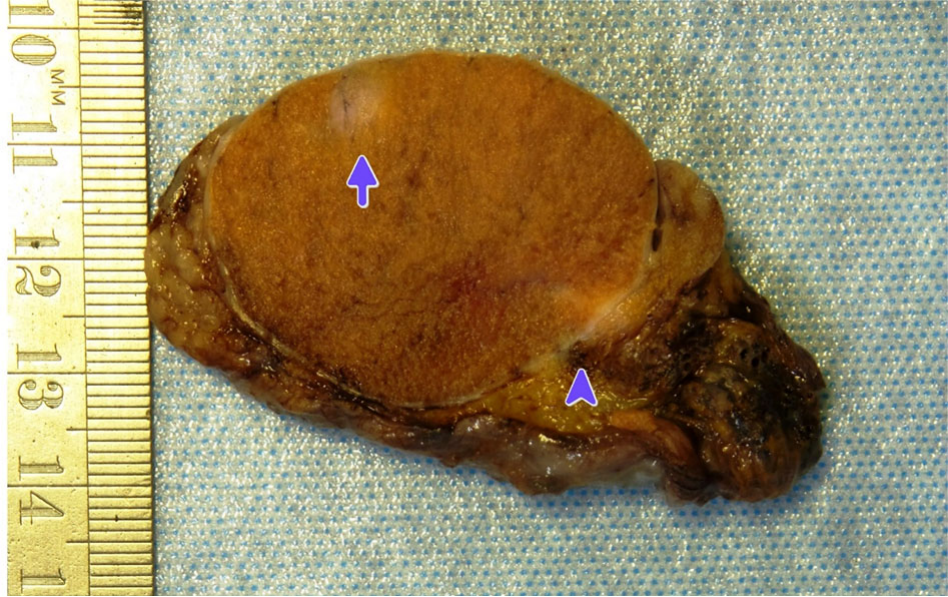
Gross photograph: One half‐bivalved radical orchidectomy specimen showing a firm area at the superior pole (arrowhead), an adenomatoid tumor on histology, and a circumscribed subcapsular tumor in the testis (arrow), an LCCSC tumor.

**Fig. 4:**
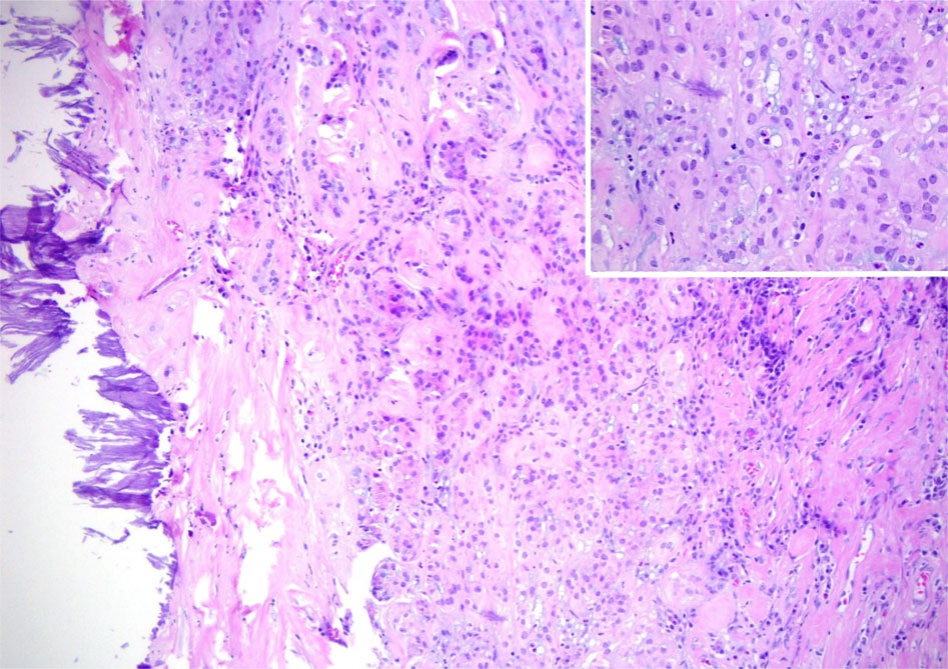
Medium power view of LCCSC tumor. Large cells with abundant eosinophilic cytoplasm, embedded in myxohyaline stroma with adjacent calcifications. Inset: High power view of tumor cells with admixed neutrophile.

On examination of the orchidectomy specimen in the pathology laboratory, two areas within the testis were targeted for sampling, a firm area adjacent to the epididymis at the superior pole and a separate 10 mm calcified area in the mid pole (Fig. 3). The superior pole showed a proliferation of calretinin and CK7 positive tubular structures consistent with an adenomatoid tumor. This correlated best with the lump detected on the exam and the ultrasound findings present in the superior pole of the testis. The separate area in the mid pole was a subcapsular tumor composed of compact tubules of eosinophilic cells, with prominent stromal hyalinization, dystrophic calcifications, and metaplastic ossification (Fig. 4). There were admixed neutrophils. There was no mitotic activity, necrosis, or vascular invasion. Immunohistochemistry showed diffuse positivity for inhibin, with focal positivity for S100, Melan‐A, and smooth muscle antibodies (SMA). Cytokeratin stains, desmin, and Oct‐4 were negative. Beta‐catenin showed cytoplasmic positivity only. This was compatible with a diagnosis of an LCCSC tumor.

This patient's post‐operative pathology and radiology were discussed at the multidisciplinary meeting, and the patient was discharged from specialist follow‐up. He is doing well 2 years postoperatively with regular self‐examinations.

## Discussion

Sertoli cell tumors are rare sex cord‐stromal testicular neoplasms, accounting for 1% of testicular tumors.^2^ LCCSC tumor is a related but distinct entity that is even rarer still, with less than 100 cases reported in the literature to date. First comprehensively described in 1980, LCCSC tumors can be benign, borderline, or malignant. Benign tumors present between 2 and 38 years, measure between 0.5–4 cm, and are more likely to be multifocal and associated with genetic syndromes. Malignant lesions occur in older patients, between 28 and 73 years, ranging from 2 to 15 cm in size.^1^

Histologically, LCCSC tumor cells are large with eosinophilic cytoplasm and myxohyaline stroma. They usually show extensive calcification. Their immunoprofile includes positivity for inhibin, S100, Melan‐A, and SMA. The nuclei are usually negative for beta‐catenin, which can help to differentiate this entity from other types of sertoli cell tumors.^3^ Features concerning malignancy are necrosis, high‐grade cytological atypia, mitotic rate >3/10 high powered field, vascular space invasion, extra‐testicular growth, and size >4 cm.^4^, ^5^ The case presented here had none of these features. However, the malignant potential is still possible without these characteristics.^4^, ^6^ A clinicopathological case series suggests LCCSC tumors with two or more adverse features receive aggressive clinical management.^3^ Spread of LCCSC tumors is usually retroperitoneal, but can be lung, liver, or bone.^6^ As this patient had no adverse features, it was felt on balance that no radiological follow‐up was required.

Ultrasound scan is the cornerstone investigation for testicular lesions. LCCSC tumors are usually well circumscribed, having multiple prominent calcifications, and hypervascularity and are regularly round and hyperechoic.^7^ Testicular microlithiasis, defined as five or more calcifications under three millimeters, are not considered to be a risk factor for malignancy. They are thought to be associated with Sertoli cell dysfunction, however, they do not prompt follow‐up on their own.^8^, ^9^

Adenomatoid tumors are a rare benign neoplasm of mesothelial origin. They are most commonly seen in the third and fourth decade of life, presenting with a painless, firm intrascrotal mass.^10^ There is no described link between adenomatoid tumors and LCCSC tumors in the literature to date. Adenomatoid tumors are a diagnosis of exclusion and cannot, in general, be differentiated from malignant lesions preoperatively.^11^ As most testicular tumors are malignant, radical orchidectomy is the standard of care. Diagnostic features on histology include tubules lined by cuboidal cells positive for cytokeratins and calretinin on immunohistochemistry.^12^

Treatment for malignant LCCSC tumors is radical orchidectomy. As there is still a paucity of data, there is no defined treatment for clinically and radiographically benign cases. As benign cases can be multifocal and bilateral, organ‐sparing surgeries have been described with good effect.^13^ LCCSC tumors associated with genetic syndromes are not uniformly benign and must be assessed on a case‐by‐case basis. Features of Carney's complex and Peutz‐Jager syndrome should be identified, and genetic testing conducted where appropriate.^13^

The case described here involved a radical orchidectomy in a young patient for two benign testicular neoplasms. Knowledge of the clinical and radiological features of benign and malignant lesions is necessary to appropriately manage these patients.

## Author contributions

Lauren Crone: Data curation; investigation; visualization; writing – original draft; writing – review and editing. Sophie C Prendergast: Visualization; writing – original draft; writing – review and editing. Nick Mayer: Supervision; visualization; writing – review and editing. Frank O'Brien: Conceptualization; project administration; supervision; writing – review and editing.

## Conflict of interest

The authors declare no conflict of interest.

## Approval of the research protocol by an Institutional Reviewer Board

Not applicable.

## Informed consent

Written informed consent was obtained by the patient for the purposes of this publication.

## Registry and the Registration No. of the study/trial

Not applicable.
